# A new extralimital sighting of *Monachusmonachus* (Hermann, 1779) in the Aquatina di Frigole NATURA 2000 site (IT9150003) beach (Salento peninsula, Apulia Region, Italy) after two decades: strategies for conservation are needed

**DOI:** 10.3897/BDJ.8.e53950

**Published:** 2020-06-15

**Authors:** Francesco Zangaro, Vincenzo Schifano, Valeria Specchia, Eftychia Tzafesta, Maurizio Pinna

**Affiliations:** 1 Department of Biological and Environmental Sciences and Technologies, Research Centre of Fishery and Aquaculture of Acquatina di Frigole, University of Salento, Lecce, Italy Department of Biological and Environmental Sciences and Technologies, Research Centre of Fishery and Aquaculture of Acquatina di Frigole, University of Salento Lecce Italy

**Keywords:** Mediterranean monk seal, *Monachusmonachus*, endangered pinniped, Citizen Science, Italian marine-coastal waters, NATURA 2000 site.

## Abstract

The Mediterranean monk seal *Monachusmonachus* (Hermann, 1779) is the most endangered pinniped in the world. In addition, its presence has not been documented for about two decades in the Apulian Region and about 10 years along the Italian coastline. In this work, we aim to describe an exceptional and well-documented observation of a subadult specimen of *Monachusmonachus* occurring during the last days of January 2020 in the Salento peninsula (Apulia Region, Italy) for the first time after two decades of local extinction in the south-western Adriatic Sea.

## Introduction

The Mediterranean monk seal (*Monachusmonachus* Hermann, 1779) is the sole representative of the genus *Monachus* (Karamanlidis et al. 2015). It is the most endangered pinniped in the world (Dendrinos et al. 2008) and one of the most threatened Evolutionarily Distinct and Globally Endangered (EDGE) mammal on Earth (Karamanlidis and Dendrinos 2015). The total estimated number of specimens is fewer than 700 individuals (Karamanlidis et al. 2015). The low population numbers, the inaccessibility of its habitat and the lack of coordinated efforts to study and protect the species resulted in the Mediterranean monk seal remaining in scientific obscurity for the greatest part of modern history (Karamanlidis et al. 2015).

Human activities are known to affect the behaviour, the abundance and the distribution of pinnipeds (Dendrinos et al. 2008). Depending on the level of human disturbance, the reactions of the endangered seal species have varied, ranging from no significant effect (Childerhouse and Gales 1998) to the abandonment of pupping sites and the active search for new breeding locations away from human activities (Dendrinos et al. 2008, Stevens and Boness 2003). Actually, in the Aegean Sea monk seals seek out pupping sites that offer them the best protection against human disturbance. Nursery habitat loss may become a limiting factor for this species, as well as the increase in human activities along the Mediterranean coasts (Dendrinos et al. 2008).

One of the highest conservation priorities for the survival of the species is the identification and protection of pupping sites with respect to increasing pressures from human development and fishing activities (Dendrinos et al. 2008).

Mediterranean monk seals have a long history of interaction with humans that includes exploitation for subsistence needs, commercial harvest and persecution as a competitor for fisheries resources or because it produced actual and perceived damage to fishing gear. Reasons for population decline in the 20th century include: increased human pressure displacing seals from their habitat; destruction/alteration of suitable habitat; continued mortality due to deliberate aggression by fishermen to eliminate a competitor (even in countries and areas where the species is legally protected); fisheries by-catch; and a mass die-off at the Cabo Blanco monk seal colony (Karamanlidis and Dendrinos 2015). Habitat deterioration, destruction and fragmentation have played a significant role in the plight of the Mediterranean monk seal. Once an inhabitant of open beaches, the species has been persecuted by humans for centuries and forced to occupy increasingly marginal habitat (Dendrinos et al. 2017). This gradual displacement process into increasingly marginal habitat (e.g. smaller and more unsuitable marine caves) has been well documented (Johnson and Lavigne 1999, González 2015). This threat is still in place today, particularly in the eastern Mediterranean (Karamanlidis and Dendrinos 2015). Population changes due to life history feature growth and reproduction responses, leading to several important changes in the functional integrity of the ecosystems and resulting in the alteration of intra- and inter-specific relationships at the community level (Orfanidis et al. 2007, Ponti et al. 2009).

Here, we describe the exceptional new sighting of a subadult specimen of *M.monachus* occurring in the Aquatina di Frigole NATURA 2000 site (IT9150003) beach (Salento peninsula, Apulia Region, Italy), after many years since local extinction in the south-western Adriatic Sea. Actually, in this area, a documented occurrence of an established population of *M.monachus* does not exist and the only sightings that have been documented are 10 years old for the Italian coasts and about 17 years old regarding the Apulian coastline (Mo 2011). During the last years, this area has mainly been affected by the presence of alien invasive species (Bariche et al. 2020).

## Methods

**Figure 1.**
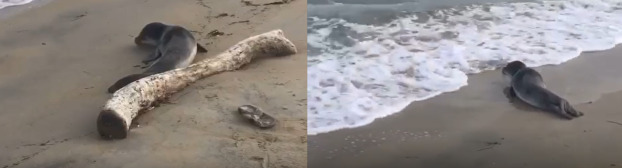
Pictures showing the new extralimital sighting of a specimen of *Monachusmonachus* in the beach of Aquatina di Frigole NATURA 2000 site (IT9150003) during the last days of January 2020.

The present observation was recorded on the 23^rd^ and 24^th^ of January 2020 (Fig. 1). The subadult specimen of *M.monachus* has been observed while resting between the beach of Aquatina di Frigole NATURA 2000 site (IT9150003) and the San Cataldo beach (40.435361°N, 18.249808°E), near to the City of Lecce (Fig. 1). The Aquatina di Frigole NATURA 2000 site is already known for the presence of the endangered bivalve mollusc *Pinnanobilis* (Linnaeus, 1758) in the lagoon (Marrocco et al. 2018).

The collection of this information and the observation of the specimen were mainly possible thanks to the reporting, the descriptions, the videos and the photos given to us by the students and the stakeholders who were present.

**Figure 2.**
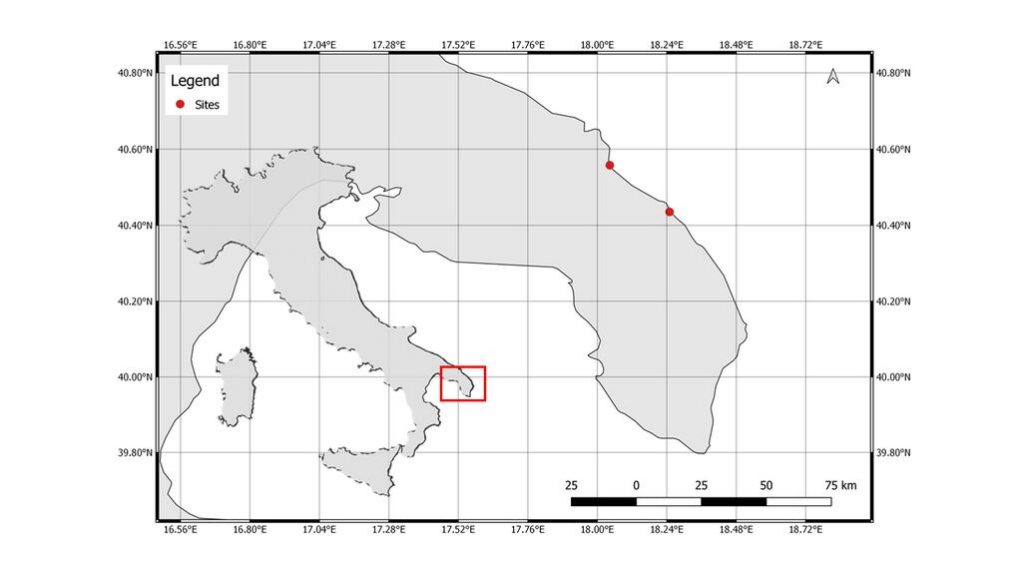
Map showing the sites along the Apulia Region coastline in which the specimen of *Monachusmonachus* was observed during the last days of January 2020.

Two days later, presumably the same specimen (the same age class and size class were described) was observed while resting on the coast of the Province of Brindisi (40.557969°N, 18.043283°E; Fig. 2), after an assumed journey of about 25 km. Here, the specimen showed signs of sickness and an ISPRA/Arpa Puglia intervention group, assisted by specialised veterinaries from the Naples zoological station, intervened on the spot in order to recover the animal (ISPRA news 2020).

Unfortunately, on the 28^th^ of January, the animal died. The body examination carried out by the Experimental Zooprophylactic Institute of Foggia (Apulia Region, Italy) showed a general state of debilitation of the animal, with impairment of the immune system combined with an inflammatory status of the respiratory tract. A strong intestinal parasitosis was also identified. (ISPRA news 2020).

## Results

**Figure 3.**
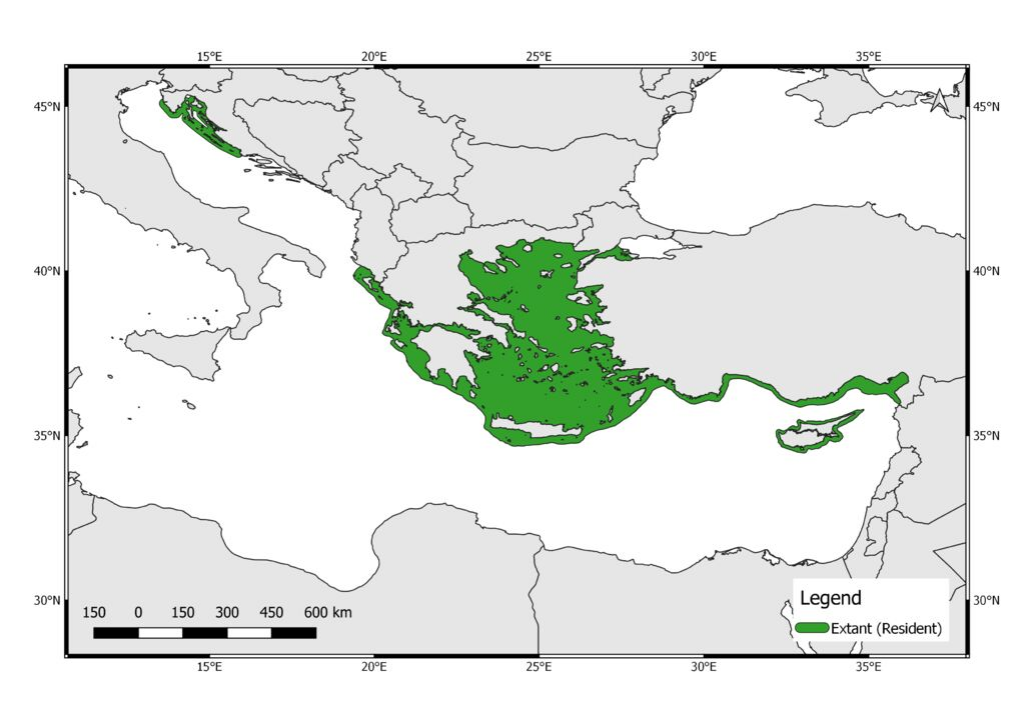
Map showing the distribution of the extant (resident) populations of *Monachusmonachus* in the Mediterranean Sea (modified from Gomerčić 2011, Karamanlidis and Dendrinos 2015).

**Figure 4.**
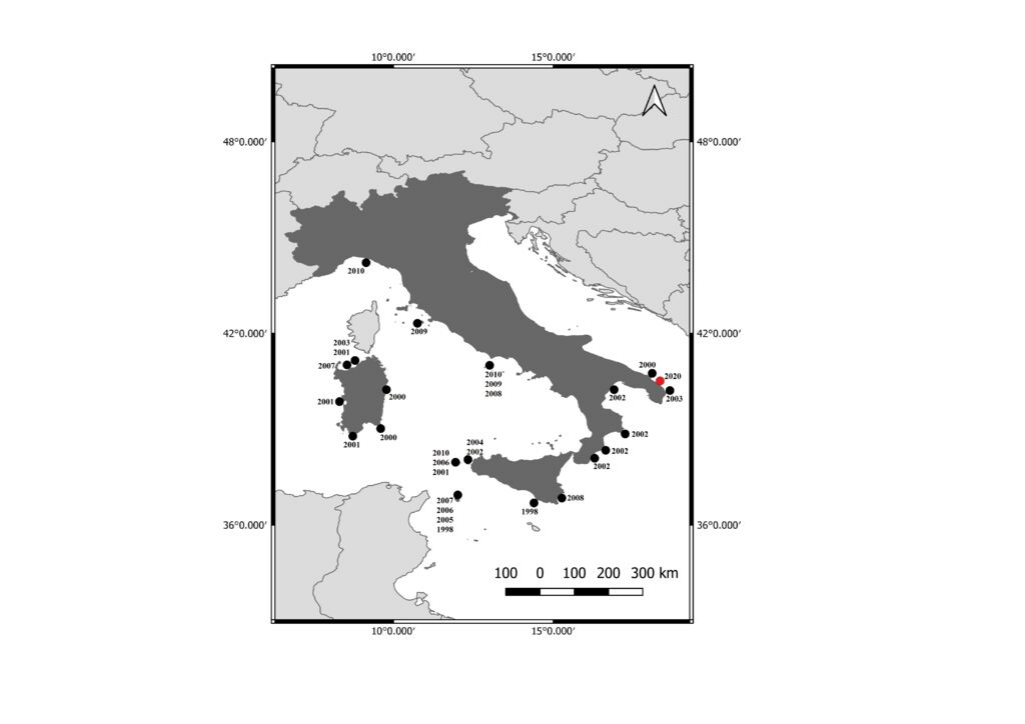
Map showing the occurrences of validated *Monachusmonachus* extralimital sightings from 1998 to 2010 in Italy (black points, Mo 2011) and the new extralimital sighting along the Apulia Region coastline (red point, January 2020).

Through the open-source software QGIS v. 3.10.0 (QGIS Development Team 2020, Bariche et al. 2020), it was possible to draw a map of the Salento peninsula showing the two areas in which the specimen occurred at the end of January 2020 (Fig. 2). The extant (resident) populations within the Mediterranean Sea are distributed in the sea's eastern reaches, around islands in the Ionian and Aegean Seas in Greece, along the mainland coasts of Greece, Cyprus and western and southern Turkey, other than along the coasts of Croatia (Gomerčić 2011, Karamanlidis and Dendrinos 2015; Fig. 3). In Italy, information about extralimital sightings of *M.monachus* is available from 1998 to 2010 (Mo 2011). A map showing the distribution and dates of occurrence of confirmed extralimital sightings of *M.monachus* (1998-2020) could be drawn (Fig. 4). Figure 4 represents a more up-to-date map on the distribution of extralimital Mediterranean monk seal sightings along the Italian coastline.

## Discussion

The geographical range of the species is highly fragmented. In the Mediterranean Sea, *M.monachus* is mainly found in the sea's eastern reaches around islands in the Ionian and Aegean Seas in Greece, along the mainland coasts of Greece, Cyprus and western and southern Turkey (Karamanlidis et al. 2015). Therefore, the more viable hypothesis is that the specimen was probably travelling from the coast of the Ionian island of Othonoi (Greece) as an extralimital dispersion. In the last ten years, other than in Italy (Mo 2011), different sightings have occurred in Israel (Scheinin 2011) and Egypt (Notarbarolo di Sciara and Fouad 2012), both areas where the species has been extinct for decades (Johnson et al. 2006). These sightings have also been attributed to animals that moved from monk seal populations in the eastern Mediterranean (Alfaghi et al. 2013).

Despite the natural death of the animal, the observation of this endangered species along the Apulian coastline is of extreme importance and it gives hope that the re-population in the Italian waters is possible. It is thus necessary to find and preserve the areas that could be the most suitable for the species life and reproduction. The beach of Aquatina di Frigole is protected under the NATURA 2000 network and the observation of such important species in the area could be considered a demonstration of good environmental management by the network.

On the other hand, the explanation for the presence of this subadult individual along the Salento peninsula coast could be found in the existence of a population already living along the Apulian coast or in the possibility that one population has been reproducing for some time in one of the caves present along the Apulian coast. This hypothesis should be followed up by intense monitoring efforts that could confirm the presence of the species in the regional waters. Moreover, monitoring efforts should be applied to identify the critical habitat for the survival of the species or the permanent recovery of a population and promote appropriate management and conservation measures (Notarbartolo di Sciara et al. 2009).

In case the individual was a migrant, it should have travelled more than 100 km in the open sea (the nearest distance from the Salento peninsula to the western coast of the island of Othonoi, Greece; Fig. 3) supporting the assumption that a Mediterranean monk seal subadult is able to travel long distances (Adamantopoulou 2011).

There is a need for studies on habitat suitability and habitat management options, as well as effective policies and interviews with people that are likely to interact with seals (fishermen, tourists and all the stakeholders). Stronger management and monitoring actions will be useful to ensure that these extralimital sightings are possible in the future in order to enhance the area, making the habitat suitable for recolonisation by this species. Moreover, extraordinary events like this demonstrate that a good level of awareness by the population is essential, resulting in the creation of a bridge between the academic world and the general audience and the creation of a Citizen Science network fundamental for the management of such exceptional events (Marrocco et al. 2019). Extralimital sightings of *M.monachus* provide important implications for future studies, in order to define the conservation measures to create a valid and reliable Citizen Science and expert information network (Adamantopoulou et al. 1999) and to create a complete catalogue of identified individuals (Marchessaux and Muller 1987, Pires et al. 2008).

Additionally, for future studies, DNA analysis should be included. DNA barcoding is a recent and revolutionary concept that refers to a single-species identification with the use of a short DNA fragment. Especially, its application on the identification of species at low densities, such as endangered or threatened ones, can prove to be very profitable. The DNA barcode of the endangered monk seal *M.monachus* can be found in the reference libraries, enabling more effective monitoring and conservation surveys (Alfaghi et al. 2013, Pawlowski et al. 2018).

In conclusion, it is necessary to raise awareness amongst stakeholders and the general public concerning the protection of Mediterranean monk seals by disseminating good practices, for the development of a viable network for the recovery of this precious species and its habitat.
